# Simultaneous bilateral quadriceps tendon rupture in a healthy young male: a case report

**DOI:** 10.1186/s13256-023-03802-7

**Published:** 2023-03-07

**Authors:** Ryo Sasaki, Masaki Nagashima, Noriyuki Aibara, Shuji Aomatsu, Shinsuke Aida, Kenichiro Takeshima, Ken Ishii

**Affiliations:** 1 Department of Orthopaedic Surgery, School of Medicine, International University of Health and Welfare, 4-3 Kōzunomori, Narita City, Chiba 286-8686 Japan; 2 Department of Orthopaedic Surgery, International University of Health and Welfare Mita Hospital, 1-4-3 Mita, Minato-ku, Tokyo 108-8329 Japan; 3 Department of Orthopaedic Surgery, International University of Health and Welfare Narita Hospital, 852 Hatakeda, Narita City, Chiba 286-8520 Japan; 4 Department of Pathology, International University of Health and Welfare Mita Hospital, 1-4-3 Mita, Minato-ku, Tokyo 108-8329 Japan

**Keywords:** Bilateral quadriceps tendon rupture, Suture anchor repair, Obesity, Young individual

## Abstract

**Background:**

Simultaneous bilateral quadriceps tendon rupture is rare, particularly in young individuals with no prior medical history. We present the case of a young man who presented with bilateral quadriceps tendon rupture.

**Case presentation:**

A 27-year-old Japanese man missed a step while descending a flight of stairs, stumbled, and became aware of severe pain in both knees. He had no past medical history, but was severely obese, with a body mass index of 43.7 kg/m^2^ (height 177 cm, weight 137 kg). Five days after injury, he was referred to our hospital for examination and treatment. Bilateral quadriceps tendon rupture was diagnosed based on magnetic resonance imaging, and quadriceps tendon repair with suture anchor was performed on both knees 14 days after injury. The postoperative rehabilitation protocol was to immobilize both knees in extension for 2 weeks, then to gradually proceed with weight-bearing and gait training using hinged knee braces. Both knees obtained a range of motion from 0° to 130° without any extension lag by 3 months postoperatively. One year postoperatively, tenderness was evident at the suture anchor in the right knee. That suture anchor was therefore removed in a second operation, and histological evaluation of the tendon of the right knee revealed no pathological changes. As of 19 months after the primary surgery, the patient showed a range of motion from 0° to 140° in both knees, did not complain of any disability, and had fully returned to normal daily activities.

**Conclusions:**

We experienced simultaneous bilateral quadriceps tendon rupture in a 27-year-old man with no past medical history other than obesity. Suture anchor repair was performed for both quadriceps tendon ruptures and achieved a favorable postoperative outcome.

## Background

Simultaneous bilateral quadriceps tendon rupture (QTR) is very rare [1], particularly among young individuals with no prior medical history. Patients with QTR experience difficulty walking due to impaired active extension of the knee and have significant functional disability. QTR requires surgical repair to avoid poor outcomes previously reported in cases of neglected or chronic rupture [2, 3]. Suture anchor (SA) repair for QTR is reported to be minimally invasive and simple in cases where the rupture site is near the patella and enables strong suturing [4]. Previous reports have identified medical histories of comorbidities, such as renal failure, rheumatoid arthritis, systemic lupus erythematosus, and hyperparathyroidism, and the use of oral steroids as risk factors for QTR [5]. In addition, elderly and obese individuals are more susceptible to this trauma [6, 7]. There are several reports of simultaneous bilateral QTR in cases with such risk factors. However, few reports of simultaneous bilateral QTR in young individuals, without risk factors for QTR, have been provided [8, 9]. We present here the case of a young man with no past medical history, other than obesity, who presented with simultaneous bilateral QTR and was treated using SAs.

## Case presentation

**Fig. 1:**
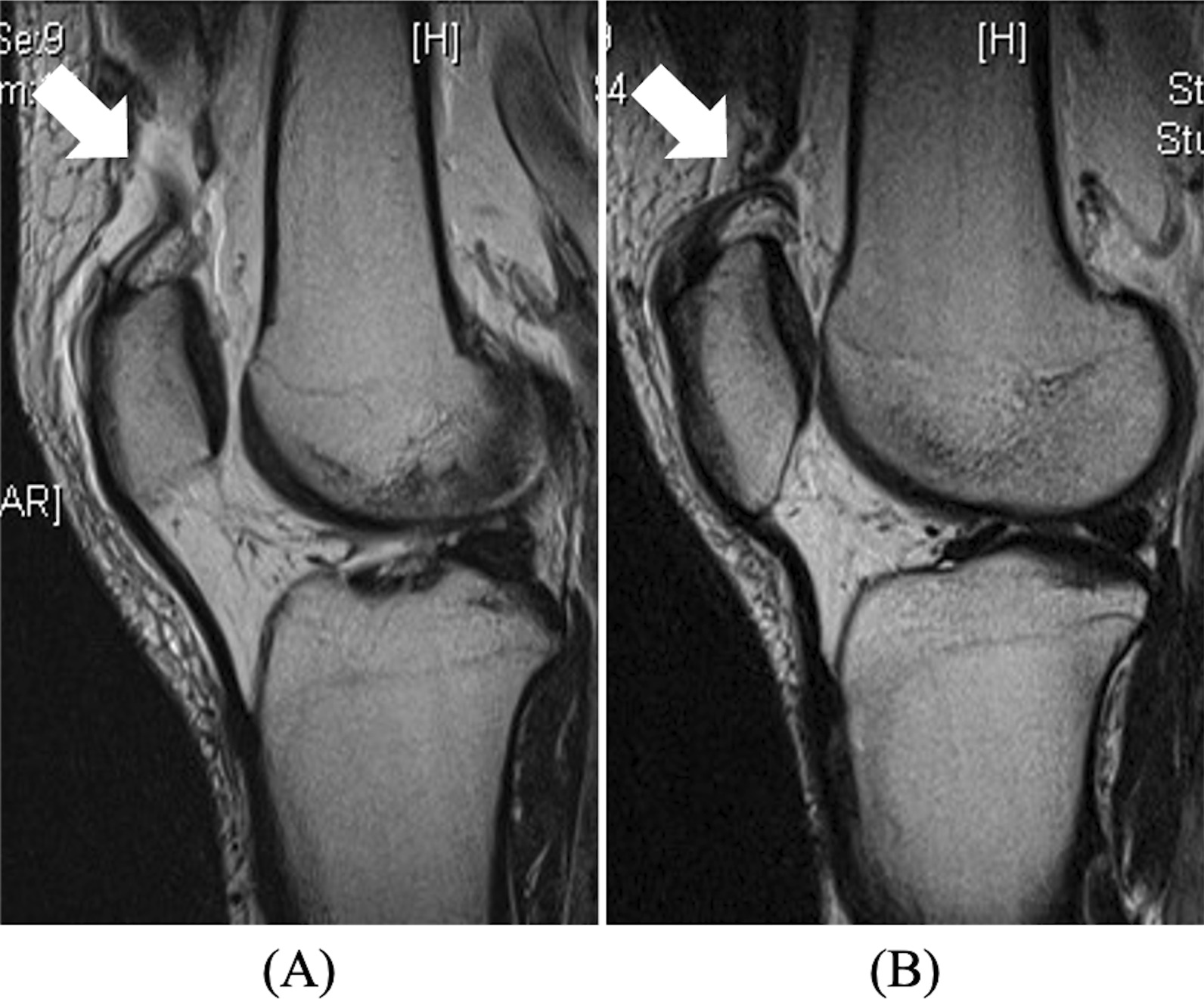
Preoperative sagittal T2-weighted imaging shows rupture of the quadriceps tendon (white arrow) of right knee (**A**) and left knee (**B**)

A 27-year-old Japanese man missed a step while descending a flight of stairs. He stumbled and both knees were forced into hyperflexion causing severe pain in both knees. He visited an orthopedic clinic complaining of inability to walk and severe bilateral knee pain. Five days after injury, he was referred to our hospital for further examination and treatment. He had no past medical history, and no history of trauma or tendonitis around both knees, but was severely obese with a body mass index (BMI) of 43.7 kg/m^2^ (height 177 cm, weight 137 kg). On physical examination, palpable defects and severe tenderness were identified around the quadriceps tendons. He was unable to actively extend either knee, and the Lysholm score was 15. Although radiographs of both knees showed no skeletal damage, magnetic resonance imaging (MRI) of both knees showed disruption of the quadriceps tendon integrity (Fig. 1).

**Fig. 2:**
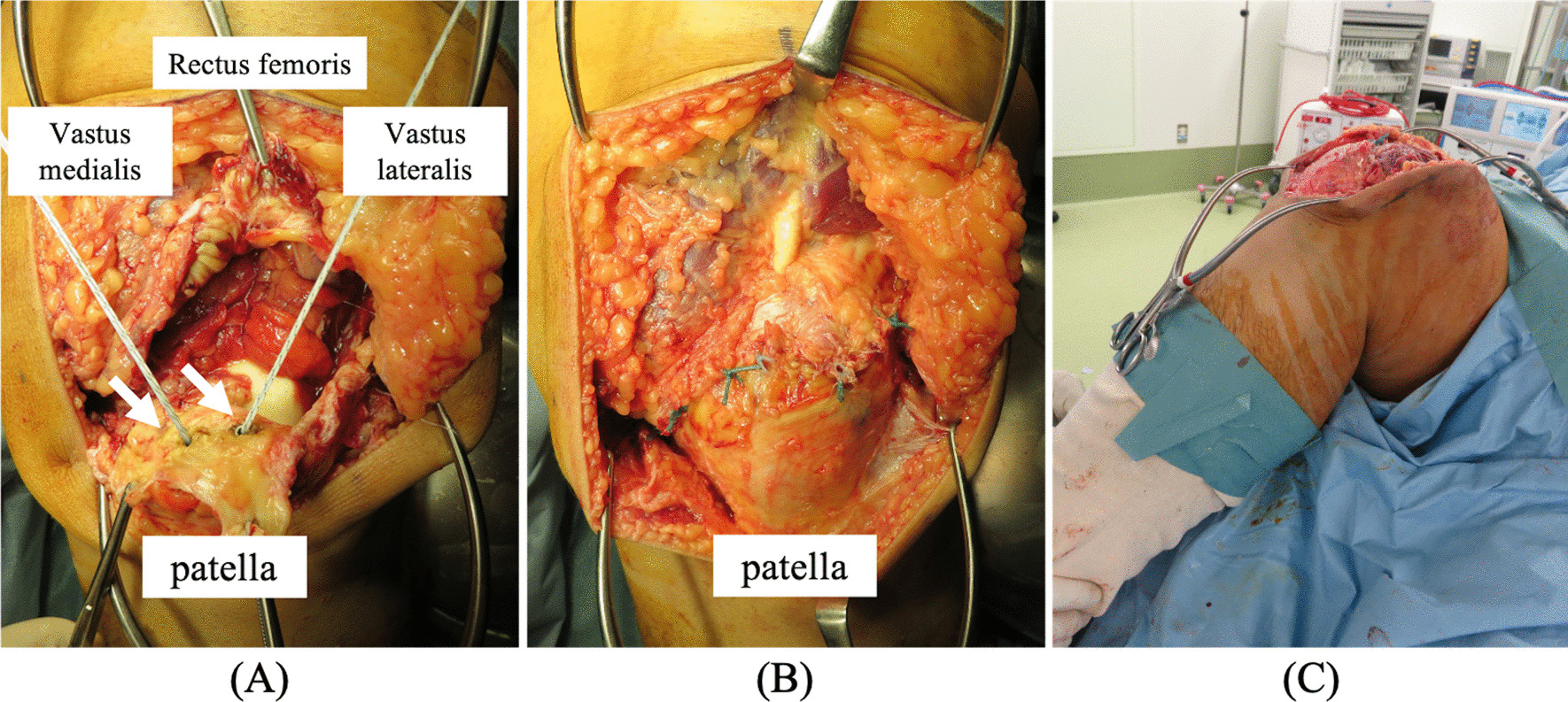
Surgical procedure for the left knee. **A** An anterior longitudinal incision almost 8 cm in length exposes the distal end of the ruptured quadriceps tendon and the proximal pole of the patella. All quadriceps tendons are ruptured at 2 cm above the superior pole of the patella. Two 5.5-mm-diameter suture anchors (Corkscrew FT; Arthrex, Naples, FL) are placed at the superior pole of the patella (white arrows). **B** The tendons are fixed end-to-end with nonabsorbable sutures passed through suture anchors. **C** The suture site is checked for instability with the knee flexed to 90°

Bilateral QTR was diagnosed based on MRI findings, and quadriceps tendon repair with SAs was performed on both knees, 14 days after injury, and under general anesthesia with tourniquet use. In the left knee, all quadriceps tendons were ruptured at 2 cm above the superior pole of the patella. The tendons were fixed end-to-end with nonabsorbable sutures passed through two 5.5-mm-diameter SAs (Corkscrew FT; Arthrex, Naples, FL) placed at the superior pole of the patella. Any additional tears were sutured appropriately with absorbable surgical sutures. The suture site was checked for instability when the knee was flexed to 90° (Fig. 2). In the right knee, quadriceps tendons, except for the vastus lateralis, were ruptured 2 cm above the superior pole of the patella, and QTR repair was performed in the same way as in the left knee. The postoperative rehabilitation protocol was to immobilize both knees in extension for 2 weeks, then to gradually proceed with weight-bearing and gait training with hinged knee braces. Both knees obtained a range of motion from 0° to 130° without any extension lag by 3 months postoperatively.

**Fig. 3:**
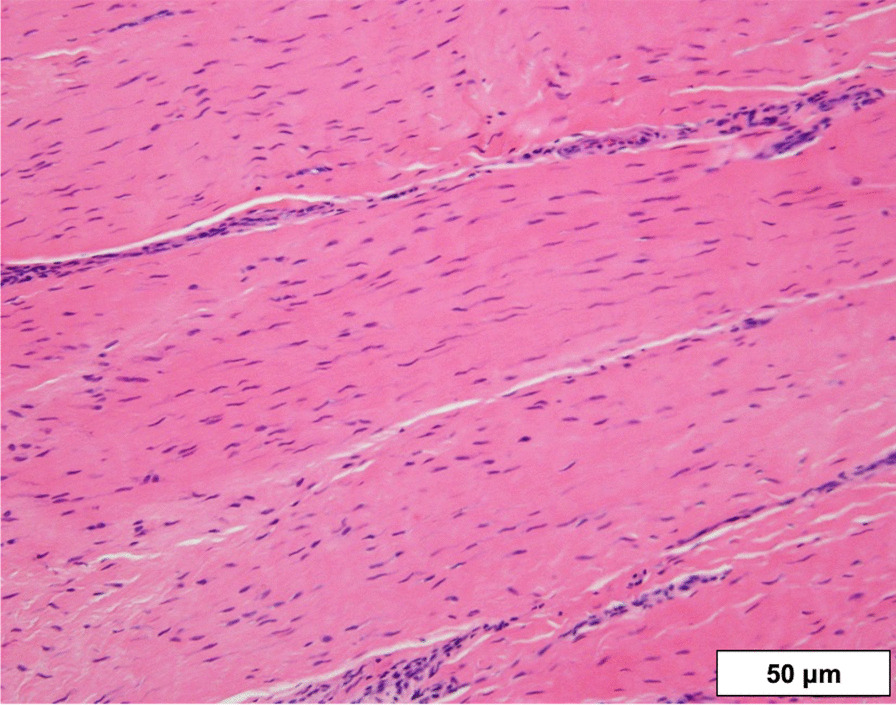
Pathological findings for quadriceps tendon taken from beside the suture anchor (hematoxylin and eosin staining). This result reveals no pathological abnormalities, other than slight inflammatory cell infiltration between tendon fibers

**Fig. 4:**
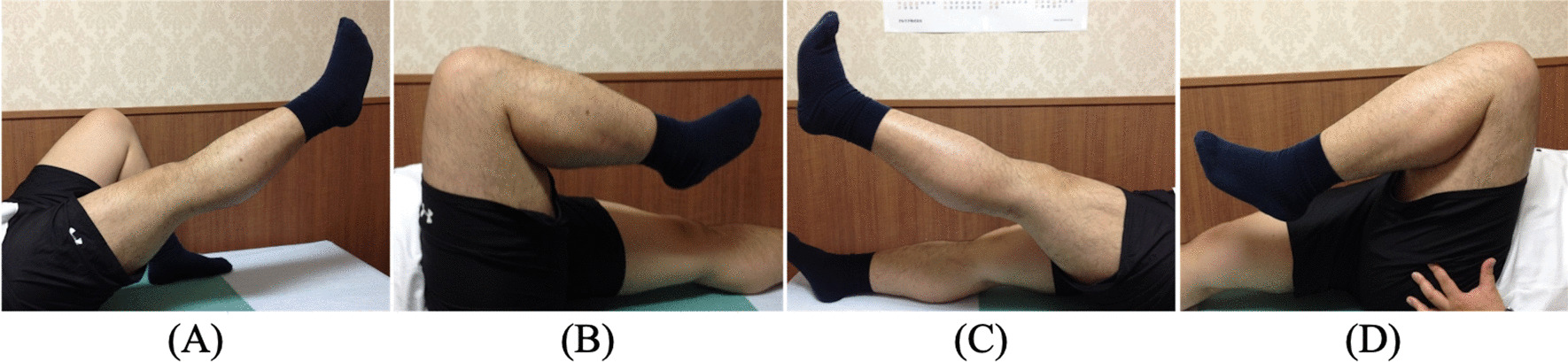
Postoperative photographs at 19 months after primary surgery for the right knee (**A**, **B**) and left knee (**C**, **D**) in extension and flexion. The range of flexion is 140°, and there is no extension lag of the knee

Twelve months postoperatively, the patient had lost 13 kg and his BMI was reduced to 39.9 kg/m^2^; however, he complained of gradually worsening right knee pain. Tenderness was evident at the superior pole of the right patella. Radiography revealed loosening of one of the SAs. Since the pain was attributed to loosened SAs, the SA placed in the right patella was removed. During this second operation, histological evaluation of the tendon of the right knee revealed no pathological abnormalities other than slight inflammatory cell infiltration between tendon fibers (Fig. 3). By 19 months after the primary surgery, the patient showed a range of motion from 0° to 140°, with no extension lag in either knee (Fig. 4). Lysholm score was 100, and the patient did not complain of any disability and had fully returned to normal daily activities. Six years after the primary surgery, no rerupture of any quadriceps tendons had occurred, and no rupture or inflammation of other tendons was identified.

## Discussion and conclusions

We encountered a very rare case of simultaneous bilateral QTRs in a 27-year-old patient with no previous medical history and no risk factors other than obesity. SA repairs were performed for both knees, and the postoperative results were excellent.

The quadriceps tendon is one of the graft choices for anterior cruciate ligament reconstruction, as it is almost as strong as the patellar tendon [10]. Unilateral QTR is a rarely reported trauma with an incidence of only 1.37 cases per 100,000 [1]. Elderly and obese individuals are known to be more susceptible to this injury [6, 7]. In addition, medical histories of comorbidities have been reported as risk factors for QTR [5]. Some studies showed that ruptured tendons usually demonstrate pathological changes, such as hypoxic degeneration, mucoid degeneration, endolipomatosis, and other types of degeneration [4, 11].

There are several reviews that have reported on simultaneous bilateral QTR [8, 9]. Those reviews indicated that bilateral QTR with no risk factors or obesity alone accounted for about 30% of all bilateral QTR. In addition, while the mean age of all bilateral QTR patients was in the early 50s, the age of bilateral QTR patients with no risk factors, or only obesity, was older—in the late 50–60s. On the other hand, the incidence of bilateral QTR in patients under 30 years of age was reported to be 10.6% (7/66 cases), and all of them had a past medical history or were taking steroids. Bilateral QTR with no past medical history, no steroid use, and no tendon degeneration among individuals under 30 years old remains extremely rare. To the best of our knowledge, this represents the youngest case with simultaneous bilateral QTR, with no past medical history other than obesity.

Minimally invasive and strong repair techniques are desirable for QTR treatment [12]. Such procedures allow early rehabilitation, which reduces patient burden (for example, hospitalization and immobilization periods) and reduces the risk of complications such as deep venous thrombosis and pulmonary embolism. One report described a case of fatal pulmonary embolism after bilateral QTR surgery [6], and elderly individuals are more likely to experience QTR and deep venous thrombosis [13, 14]. Favorable surgical outcomes have also been described with the previously reported technique of reattaching the tendon through bone tunnels, with or without tendon augmentation, such as artificial tendon, wire, or fascia, although these methods have been shown to be invasive and complicated [15, 16]. In contrast, SA repair requires only a small incision from the superior pole of the patella to the area of tendon rupture, and is less invasive, while still permitting strong repair of the ruptured tendon [17], regardless of the margin available for sewing. A recent cadaveric study reported no statistically significant difference in the ultimate load to failure between SA repair and reattaching the tendon through bone tunnels [18, 19]. Clinical studies have reported successful outcomes from SA repair [4]. Given such findings, we performed SA repair in this case, and achieved favorable clinical outcomes.

In conclusion, we experienced simultaneous bilateral QTR in a 27-year-old man with no past medical history other than obesity. SA repair was performed for both QTRs and achieved a favorable postoperative outcome.

## Data Availability

The datasets of the present study are available from the corresponding author upon reasonable request.

## Abbreviations

QTR: Quadriceps tendon rupture
SA: Suture anchor
BMI: Body mass index
MRI: Magnetic resonance imaging
